# Helicobacter pylori in colorectal neoplasms: is there an aetiological relationship?

**DOI:** 10.1186/1477-7819-5-51

**Published:** 2007-05-12

**Authors:** Mary Jones, Peter Helliwell, Colin Pritchard, Joseph Tharakan, Joseph Mathew

**Affiliations:** 1 Department of Histopathology, Royal Cornwall Hospital, Truro, TR1 3LJ, UK; 2 Department of Research and Development, Royal Cornwall Hospital, Truro, TR1 3LJ, UK; 3 Department of Medicine, Princess Alexandra Hospital, Harlow, Essex, UK

## Abstract

**Background:**

This pilot study was carried out to determine whether *Helicobacter pylori *can be detected in normal colon or in association with colorectal neoplasia.

**Methods:**

Paraffin processed colonic tissue blocks of normal colonic mucosa (n = 60), and patients diagnosed as adenoma (n = 60), and adenocarcinoma (n = 60) were retrieved from our archive; the adenoma group included tubular (n = 20), tubulovillous (n = 20) and villous adenomas (n = 20). 4 μm sections were stained by immunohistochemical methods using anti-*Helicobacter pylori *antibodies (polyclonal NCL-HPp and monoclonal NCL-C-jejuni).

**Results:**

Significant numbers of *Helicobacter pylori *were identified in tubular adenomas (OR = 11.13; 95%CI = 1.62–76.70), tubulovillous adenomas (OR = 10.45; 95%CI = 1.52–71.52) and adenocarcinomas (OR = 8.13; 95%CI = 1.40–46.99) compared to controls: there was no association in numbers of *Helicobacter pylori *and villous adenomas (OR = 2.95; 95%CI = 0.29–9.96).

**Conclusion:**

We conclude that although, in this pilot study, there appears to be an association in the prevalence of *Helicobacter pylori *with some, but not all, colorectal neoplasms, we can not infer causality from these results. These findings need to be further substantiated with a prospective study and the use of molecular biological techniques to determine a causal association.

## Background

Colorectal cancers develop sequentially from hyperproliferative epithelium and aberrant crypt foci through to adenocarcinomas, passing through an adenomatous stage [1,2]. These adenomatous foci are neoplastic intraluminal protuberant, occasionally flat, dysplastic glandular lesions, represented by tubular or villous adenomas at either architectural extreme [3]; aetiologically, environmental, social and genetic factors or influences have been implicated in their genesis [4-7]. The possibility however of *Helicobacter pylori *(HP) as an initiator of colorectal neoplasia [8,9], similar to its role in gastric carcinoma and lymphoma [3,10] is subject of investigation. It is well established that HP is associated with extragastric disease [11], as are several non-HP species [12,13]; indeed HP has been identified outwith the stomach [11,14-16], in the intestine [14,15,17] as well as in faeces [18]. Most associations between neoplastic colorectal lesions (adenomas and carcinomas) and HP are based on studies correlating these lesions with HP seropositivity [9,19-23] or, indirect evidence such as increased gastrin [24,25] or CagA+ levels [24]. Other studies have failed to demonstrate this association based on seropositivity [26-28]; indeed it has been suggested that HP does not colonise rectal mucosa [29].

In this study we have used immunohistochemical methods to interrogate normal, adenomatous colorectal tissue and colorectal adenocarcinomas for the presence of HP, using anti-HP antibodies.

## Methods

This pilot study was independently examined and ethically approved by the Local Research Ethics Committee, Royal Cornwall Hospital, Cornwall, UK.

Samples of paraffin-embedded colorectal tissue (n = 180) including normal (n = 60), adenomas (n = 60) and adenocarcinomas (n = 60) were retrieved from departmental archives. Sixty samples of each diagnosis gave an acceptable precision for the prevalence estimates. This powered the study at 0.80 (α = 0.05) to detect an absolute difference of 26% in the prevalence of the different diagnoses. Specimens were not matched for age, sex or socioeconomic status as it was thought that this would confound any comparisons; a higher prevalence in adenocarcinomas might simply reflect the greater age of the people with this type of neoplasia. However, the age and sex were adjusted for using binary logistic regression once the results had been established. The number of males and females and their mean ages in each group is shown in Table 1. Patients included in the normal category had biopsies for non-specific gastrointestinal symptoms, iron deficiency investigation or diarrhoea, but whose histology was unremarkable.

**Table 1:** Histological prevalence of HP and demographics of patients with colorectal neoplasms and controls

**Pathology diagnosis**	**Adenocarcinoma**	**Adenoma**			**Normal (Controls)**
		**Villous**	**Tubulovillous**	**Tubular**	
Number of cases	59	20	20	19	58
Male:Female	22:37	9:11	13:7	14:6	25:33
Mean age (*range*)	67.55 (*36–91*)	73.85 (*55–94*)	69.45 (*47–84*)	66.75 (*44–84*)	51.60 (*22–86*)
HP prevalence (*%positive*)	10/59 *16.9*	1/20 *5*	4/20 *20*	4/19 *21*	1/58 *1.7*
Odds ratio (OR) (*95% CI*)	8.13 (*1.40–46.99*)	2.95 (*0.29–9.96*)	10.45 (*1.52–71.52*)	11.13 (*1.62–76.70*)	
Age-sex adjusted Odds Ratio (*95% CI*)	8.73 (*1.01–75.48*)	1.94 (*0.10–36.77*)	5.73 (*1.02–112.83*)	11.53 (*1.12–118.98*)	

Each sample group of patients was separate and mutually exclusive. Patients with normal biopsies did not have a biopsy history of colorectal adenomas or carcinomas, or other carcinoma. The patient group with adenomas did not have a biopsy history of colorectal or other carcinoma. Patients with colorectal adenocarcinoma formed the third group: these patients did not have a biopsy history of cancer at any other site. The limitations of this study include unavailability of information relating to serology or breath test or, preceding gastric biopsy.

Since there is a sequential progression of colorectal polyps to colorectal adenocarcinoma, 20 cases each of tubular, tubulovillous and villous adenomas were selected. Immunohistochemical techniques were chosen because they are more specific and sensitive than tinctorial techniques [30-33].

Cases were identified from the histopathology database between 1996 and 2001, using appropriate T and M codes and the relevant paraffin blocks were retrieved. Topographical code T67*/68* identified biopsies from the colorectum. Morphology code M00100 identified biopsies considered normal; M80400, M82630, M82611 and M81403 detected adenomas and carcinomas.

Four 4 μm sections were cut from each of the 180 formalin fixed paraffin blocks using a Leica^® ^rotary microtome. Diagnosis were confirmed with an H/E stain; the other sections were used for immunohistochemistry. H/E and immunohistochemistry were used to detect HP organisms; Giemsa was not used. The immunohistochemically stained slides were randomized, given individual research numbers and then examined by light microscopy

Two Novocastra^® ^antibodies were used: 1) polyclonal NCL-HPp (specific and sensitive for HP; dilution 1:150)[34,35] and, 2) monoclonal NCL-C-jejuni (raised against *campylobacter *jejuni; dilution 1:800), which cross-reacts with HP. The use of two antibodies allowed for identification and differentiation of HP from Campylobacter in our study material. The strategy was to see whether we could identify one or the other, using immunohistochemistry on protocol samples.

Antigen retrieval was carried out in a citrate buffer pH6.0 for 30 minutes at 430W in a domestic microwave. This method was chosen over the Novocastra recommended trypsin digestion following experiments comparing both methods, as recommended[34,35]. Positive controls were gastric biopsies known to be positive for HP; the primary antibody was omitted in negative controls.

The detection method followed was that provided by Dako with the ChemMate™ Envision detection kit [36]. The ChemMate™ Envision detection kit was used as the dextran polymer based secondary antibody technique is a two step process which is fast, reliable and with superior sensitivity to the avidin-biotin method [36].

Sections were counterstained using Mayer's Haematoxylin for 1 minute and blued in Scotts Tap water. Odds ratios (OR) were estimated using the Logit procedure in SPSS11.

## Results

Four cases were excluded from this analysis; two from the normal tissue group, one from the adenocarcinoma group and one from the tubular adenoma group. These were due to inadequate tissue samples.

**Figure 1:**
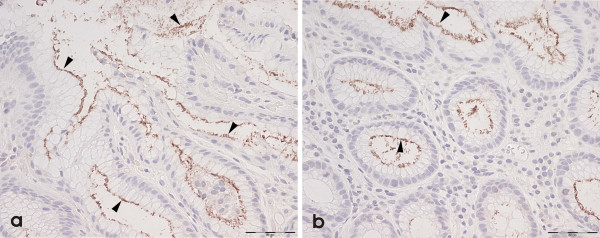
Positive HP immunohistochemistry (arrowheads) is seen in gastric control material using NCL-HPp (a & b) (internal scale = 50 μm).

**Figure 2:**
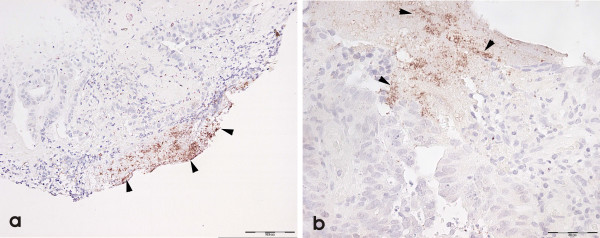
Immunopositivity is seen in colorectal neoplasia using a) NCL-HPp and b) NCL-C-Jejuni (internal scale = 50 μm).

HP organisms were golden-brown "dot-like" and granular against a light blue Haematoxylin counterstain. We were unable to unequivocally detect the spiral form of HP on H/E or immunohistochemistry in any of our normal or neoplastic colonic biopsy samples; we assume from this that the "granular" HP-positive organisms represented the coccoid form [37-39] of the organism. Figure 1 shows positive anti-HP immunohistochemical reactions in control gastric mucosa with NCL-HPp (Figure 1a &1b). Figure 2 shows colonic mucosa with positive anti-NCL-HPp (Figure 2a) and anti-NCL-C-jejuni (Figure 2b) immunohistochemistry. The positivity with both antibodies was convergent; we did not observe positivity of one antibody without the other also being positive. We concluded from this that we were observing only HP organisms, rather than non-HP organisms or other Helicobacter species. These organisms were luminal[32,40] although a rare organism was seen in a crypt. Their staining patterns were similar to that in the stomach although in the latter, organisms were frequently seen in glandular lumina.

The patient demographics and results are listed in table 1. There were more females in each group, with the exception of patients with tubular and tubulovillous adenomas. HP was detected in only one normal (control) (2%) specimen but between 17 and 21% of patients with colorectal neoplasia; only 5% of patients with villous adenomas showed immunoreactivity for HP organisms. Compared to normal (controls) therefore, significant numbers of HP are seen in tubular (OR 11.13; 95% Confidence interval (CI) 1.62–76.60) and tubulo-villous (OR 10.45; 95% CI 1.52–71.52) adenomas and, in adenocarcinoma (OR 8.13; 95% CI 1.40–46.99) but not in villous adenomas (OR 2.95; 95% CI 0.29–9.96).

## Discussion

We have used immunohistochemical methods to demonstrate that HP organisms do reside in the colorectum associated with colorectal neoplasms. Although we can not attribute a cause-and-effect relationship in this instance, there is certainly indirect evidence demonstrating this association[9,19-25,41,42], as there is evidence refuting it [26-29]. Clearly, the use of serum-based methods can lead to ambiguous results especially since antibodies can be detectable a long time after the bacteria have ceased to colonise the gut[28].

We used immunohistochemical detection methods rather than routine histochemistry (Giemsa) as the former are said to be more accurate[32] and are more likely to detect non-spiral forms of the organism[31,33,37-39]. However Giemsa has been reported to be more sensitive for the detection of spiral gastric forms of the organism[43].

Our results show that the prevalence of HP is significant in tubular and tubulovillous adenomas but not in villous adenomas; the reason for this is unclear. It may be that the microenvironment in villous adenomas does not support HP in a similar manner to absence of HP in foci of intestinal metaplasia in the stomach[5] or, low HP prevalence is some types of gastric fundic polyps[44]. The absence of HP in association with villous adenomas does not necessarily signify an absence of an association but merely that HP could have migrated away from these polyps after having initiated the lesion (if indeed there is such a relationship). This would be analogous to HP migration having first produced gastric abnormalities[45].

Although some studies have not been able to substantiate an association between HP and colorectal neoplasia and HP [26-28] or, that these organisms can colonise the colon[29,46], we have been able to demonstrate their presence (using immunohistochemistry) in certain types of colorectal neoplasia; we have not looked at HP in association with other non-neoplastic epithelial lesions. Indeed, in one study, 27% of colorectal adenocarcinomas contained *Helicobacter *DNA; however, this study could not determine a statistical association between its presence and the different Dukes stages of colorectal adenocarcinomas or, between colonic and rectal adenocarcinomas[41].

HP-induced carcinogenesis is thought to be initiated by gastrin-induced genomic instability (gastrin hypersecretion being promoted by the antral effects of HP), its vacuolating toxin VacA [47-50], and upregulation of COX-2[24,51]. Most of these studies have been able to define this relationship based on serological data but does not address direct colonisation of colorectal mucosa as a necessary ingredient in the evolution of colorectal neoplasia; indeed these theories define remote activation of colorectal neoplasia by upstream HP activity and effects. Could, for instance, in some circumstances, remote and local effects synergise to initiate or produce these neoplasms[42,52]?

Experimentally, 129/SvEv Rag-2-deficient mice have been shown to develop colitis and colonic cancer in the presence of *Helicobacter. hepaticus*[53], with implications relating to immune regulation in this process. More recently, colonic adenocarcinomas have been initiated in SMAD3-deficient mice that have been exposed to *Helicobacter *infection suggesting a causal association[42].

Whereas we acknowledge that the presence of HP in our study does not necessarily mean that local HP effects are responsible for colorectal neoplasia, but neither does it exclude it. It is entirely possible also that these HP organisms are transiting the bowel and their localisation in colorectal neoplasia, fortuitous. We have also considered the possibility these results could reflect identification of HP or HP-like antigens in luminal debris: whist we can not exclude this, our anti-HP antibody is HP-specific[54]. Clearly this pilot study needs to be progressed to molecular biological techniques such as the polymerase chain reaction (PCR); PCR however is said to be no more sensitive than routine histopathological or microbiological techniques[32]; these methods require stringency in the collection of diagnostic material[32].

## Conclusion

We have been able to identify HP organisms in colorectal neoplasms using immunohistochemical methods in this pilot study. We have also been able to demonstrate an association in their presence with tubulovillous and tubulovillous adenomas and adenocarcinomas, but not with villous adenomas. These findings need to be further substantiated with a prospective study and the use of molecular biological techniques to determine a causal association.

## Competing interests

The author(s) declare that they have no competing interests.

## Authors' contributions

This manuscript is the essence of the MSc Thesis of **MJ**. The idea for this research was developed by **JT**, **JM**, **PH **and **MJ **with **CP **providing the statistical validities before, during and after the study. All slides were reviewed by **MJ**, **PH **and **JM **before and after immunohistochemistry. The immunohistochemistry was optimised by **MJ**. Randomisation of cases was done by a colleague. The authors have been variously but actively involved in its conception and design, analysis and interpretation of data, in the preparation and critical evaluation of its content, have read and approved the final manuscript.

## Financial support

We acknowledge contributions from the following sources

1. Cellular Pathology Research Funds (JM is a co-signatory)

2. Sunrise Appeal

3. Silverfern Research.
